# The association between circulating irisin levels and osteoporosis in women: a systematic review and meta-analysis of observational studies

**DOI:** 10.3389/fendo.2024.1388717

**Published:** 2024-08-08

**Authors:** Xiaoyang Shen, Yan Chen, Jing Zhang, Meina Yang, Lu Huang, Jiaqi Luo, Liangzhi Xu

**Affiliations:** ^1^ Reproductive Endocrinology and Regulation Laboratory, West China Second University Hospital, Sichuan University, Chengdu, China; ^2^ Department of Obstetrics and Gynecology, West China Second University Hospital, Sichuan University, Chengdu, China; ^3^ Key Laboratory of Birth Defects and Related Diseases of Women and Children, Ministry of Education, Sichuan University, Chengdu, China

**Keywords:** irisin, osteoporosis, bone mineral density, fracture, meta-analysis

## Abstract

**Objective:**

This systematic review and meta-analysis aimed to investigate the association between circulating irisin levels and osteoporosis in women, exploring irisin’s potential role in the pathophysiology and management of osteoporosis.

**Method:**

We searched PubMed, Embase, Web of Science, Cochrane Library, CNKI, WanFang, and VIP databases up to January 2023. The inclusion criteria were observational studies reporting on circulating irisin levels in women. The standardized mean difference (SMD) and correlation coefficients with a 95% confidence interval (CI) were used as the main effect measures under a random-effects model. Heterogeneity was evaluated using the Cochrane Q statistic and the *I^2^* statistics. Subgroup analysis and univariate meta-regression analysis were performed to identify the sources of heterogeneity. The quality of the included study was assessed by the Newcastle-Ottawa Score. The quality of evidence was evaluated using the GRADE system. Publication bias was assessed using Begg’s and Egger’s test, and the trim-and-fill method. Sensitivity analysis was performed to assess the stability of the results.

**Results:**

Fifteen studies with a total of 2856 participants met the criteria. The analysis showed significantly lower irisin levels in postmenopausal osteoporotic women compared to non-osteoporotic controls (SMD = -1.66, 95% CI: -2.43 to -0.89, *P* < 0.0001; *I^2^* = 98%, *P* < 0.00001) and in postmenopausal individuals with osteoporotic fractures than in non-fractures controls (SMD = -1.25, 95% CI: -2.15 to -0.34, *P* = 0.007; *I^2^* = 97%, *P* < 0.00001). Correlation analysis revealed that irisin levels positively correlated with lumbar spine BMD (r = 0.37, 95% CI: 0.18 to 0.54), femoral BMD (r = 0.30, 95% CI: 0.18 to 0.42), and femoral neck BMD (r = 0.31, 95% CI: 0.14 to 0.47) in women. Despite significant heterogeneity, the robustness of the results was supported by using the random effects model and sensitivity analysis.

**Conclusion:**

The current evidence suggests that lower irisin levels are significantly associated with osteoporosis and fracture in postmenopausal women, suggesting its utility as a potential biomarker for early detection of osteoporosis and therapeutic target. However, further high-quality prospective research controlling for confounding factors is needed to clarify the relationship between irisin levels and osteoporotic outcomes.

**Systematic review registration:**

https://www.crd.york.ac.uk/PROSPERO, identifier CRD42023410264.

## 1 Introduction

Osteoporosis (OP) is a pathological condition characterized by decreased bone mass, microstructural deterioration of bone tissue, and reduced bone strength, which significantly increases the risk of fractures (1). OP poses a substantial public health challenge across the world. Notably, postmenopausal osteoporosis (PMOP) is a major type of primary osteoporosis, which is particularly prevalent among women over 50 years of age, resulting from estrogen deficiency after menopause. PMOP affects nearly half of this population, a statistic that is particularly concerning in the context of an aging global population (2). PMOP and its serious complication of fracture significantly impact the mortality rate and quality of life of older women, while imposing considerable global health and economic burdens. For instance, low bone mineral density (BMD) has led to a rapid increase in disability-adjusted life-years and deaths in women globally, with a rise of 101.44% and 116.87% respectively compared with the data in 1990 (3). In 2019, the total direct cost of osteoporosis and the 4.3 million fragility fractures it caused in the European Union plus the UK and Switzerland amounted to €56.9 billion (4). Consequently, the early detection and diagnosis of OP are critical to mitigate the associated health risks, especially in postmenopausal women.

The most widely validated technique to measure BMD is dual-energy X-ray absorptiometry (DXA) at various skeletal sites. However, the high cost and limited accessibility impede its applications for widespread use and primary care inspection. Blood markers, which can detect changes in bone metabolism earlier than DXA and provide insights into bone formation and resorption dynamics without exposing patients to radiation, offer a more frequent, risk-free alternative. Despite their widespread use in clinical settings, classical biomarkers like C-terminal telopeptide of type I collagen (CTX-I) and procollagen type 1 N-terminal propeptide (P1NP) have some limitations, such as low specificity (due to synthesis in tissues other than bone), susceptibility to circadian changes, fasting and feeding, and renal function (5, 6). To address these challenges, recent research has focused on the exploration of novel biomarkers reflecting specific biological processes in bone for diagnostic, prognostic, and monitoring purposes. These emerging biomarkers may provide an accessible alternative to conventional imaging examinations and existing biomarkers.

Irisin, discovered in 2012, a novel myokine encoded by the FNDC5 gene has garnered attention under the trend. Approximately 72% of circulating irisin is derived from skeletal muscle during exercise (7), and it is widely present in various body tissues, including the pancreas, testes, liver, and stomach (8). Irisin has been implicated in the regulation of various endocrine and metabolic disorders, such as obesity and insulin resistance (9, 10). Numerous clinical studies have demonstrated the correlation between serum irisin levels and bone health indicators in different populations. For example, Colaianni reported that irisin levels positively correlated with BMD in older adult patients (11). In addition, many clinical studies have indicated decreased irisin levels in postmenopausal women with osteoporosis and a correlation between lower irisin levels and an elevated risk of osteoporotic fractures (12–14). These findings suggest that irisin could serve as a predictor and prognostic biomarker for OP. Further experimental studies have supported irisin’s significant role in bone metabolism and remodeling. For instance, irisin increased cortical bone mass in rodents and enhanced bone mass in ovariectomized mice, a model for PMOP studies (15, 16). Given the evidence from both human and experimental studies, irisin-based therapies could be promising targets for the treatment of osteoporosis. Furthermore, decreased estrogen levels in postmenopausal women increase the incidence of metabolic diseases, which are established risk factors for osteoporosis (17). Moreover, studies have also linked reduced irisin levels to sarcopenia in postmenopausal women, a known risk factor for fractures (18, 19). Overall, these multiple benefits of irisin on metabolic and bone health make it particularly valuable for addressing the multifaceted health challenges faced by postmenopausal women.

Although the positive impact of irisin on bone metabolism has been widely researched, recent studies have yielded conflicting findings regarding irisin’s association with BMD. Some studies reported decreased irisin levels in postmenopausal women with OP or fractures (12–14), while others found no significant correlation (20, 21). Similar inconsistencies are noted among premenopausal women regarding the relationship between irisin and BMD or Z scores (22, 23). Therefore, whether menopausal status might influence the relationship between irisin and bone health remains unclear. This inconsistency might stem from the heterogeneity in study populations, such as variations in age, Body Mass Index (BMI), and other factors affecting bone metabolism. Another source of inconsistency is the clinical trial design heterogeneity, such as age-matching, and the lack of uniformity in the diagnostic criteria for osteoporosis. A meta-analysis published in 2019 by Zhou et al. indicated a reduction in irisin levels in postmenopausal women, yet this study had several limitations (24). Firstly, its inclusion of both genders created heterogeneity because sex differences between men and women resulted in different metabolic states. In addition, the prior meta-analysis only included older populations and lacked thoroughly examining premenopausal women. Meanwhile, the effect of hormone levels on the relationship between irisin and BMD was not investigated. Furthermore, its statistical methodology, which used the mean difference (MD) as the effect size to pool studies with different kits, may have led to the pooling of non-comparable data, thereby rendering the meta-analysis results less meaningful. Finally, the previous meta-analysis did not conduct a subgroup analysis of age, BMI, and other potential confounding factors to identify the sources of heterogeneity.

Given the significance of this issue, the discrepancies in existing findings, and the limitations of previous meta-analysis, we conducted this systematic review and meta-analysis to further elucidate the relationship between irisin and osteoporosis/fractures/BMD in women. This present study, which includes recent research on both postmenopausal and premenopausal women, is the first to analyze the influence of hormone levels on the relationship between irisin and BMD. By using standardized mean difference (SMD) as the effect size and conducting subgroup analysis and meta-regression, we included a larger and more diverse sample size, standardized results across different measurement techniques, and provided insights into irisin’s potential applications in preventing and treating osteoporosis at various stages of a woman’s life.

## 2 Methods

We conducted this meta-analysis following the Preferred Reporting Items for Systematic Reviews and Meta-analysis (PRISMA), based on a protocol registered in the International Prospective Register of Ongoing Systematic Reviews (PROSPERO) system (ID: CRD42023410264) (25).

### 2.1 Search strategy

We searched four English electronic databases (PubMed, Embase, Web of Science, Cochrane Library) and three Chinese electronic databases (the Chinese National Knowledge Infrastructure (CNKI), WanFang, and VIP). Searches were conducted using the following MeSH key terms and word variants: (‘osteoporosis’ OR ‘Bone Density’ OR ‘Fractures, Bone’) AND (‘irisin’ OR ‘FNDC5’). The search covered records from database inception up to January 2023. The detailed search strategy is provided in **Supplementary File 1**.

### 2.2 Study eligibility and selection

Two independent authors evaluated and selected relevant studies based on the following criteria:

1) Population: postmenopausal women or premenopausal women.

2) Outcomes: studies that provide data on at least one of the outcomes: comparing the circulating levels of irisin in OP and non-OP groups; comparing the circulating levels of irisin in fracture and non-fracture groups; analyzing the correlation between circulating irisin levels and BMD at the lumbar spine or femoral or femoral neck.

3) Study type: observational studies including cross-sectional, case-control, and cohort studies.

4) The language was English or Chinese.

Exclusion criteria:

1) animal studies, conference abstract, review, and case reports.

2) the full text of literature could not be obtained.

3) the original data of the study could not be converted or used.

4) did not distinguish sex or menopausal status.

Any disagreements were resolved by consultation with a third reviewer (JZ).

### 2.3 Data extraction

Two researchers (XYS and MNY) independently extracted the following information based on the registered protocol and Cochrane handbook through a standardized Excel spreadsheet. Data extracted from each article comprised: 1) study characteristics: title, name of first author, year of publication, country, type of study; 2) basic characteristics of individuals including sample size, mean age, mean BMI, fasting status, bone densitometry method and site, fracture site, definition of osteoporosis; 3) details of irisin examination including assay kit manufacturer and mean irisin concentration and respective standard deviation (SD); 4) correlation coefficients between irisin levels and BMD. We extracted data from healthy controls, excluding any individuals with the disease under study. Any disagreements were resolved by consultation with a third reviewer (JZ).

### 2.4 Assessment of the methodological quality and quality of evidence

Two reviewers (XYS and MNY) independently assessed the methodological qualities of the included studies using the Newcastle-Ottawa Scale (NOS) (26). The NOS grades the study a maximum of nine points based on three quality aspects: the selection of the study groups, the comparability of the groups, and the ascertainment of the outcome of interest. The total score of > 7, 5–6, and < 5 were considered high-, medium-, and low methodological quality respectively (27).

The quality of the evidence for each outcome was evaluated by two authors (XYS and MNY) using the Grading of Recommendations Assessment, Development, and Evaluation (GRADE) tool independently (28). Following the GRADE principle, observational research initiated with low-quality evidence may be subjected to downgrades in terms of five factors: risk of bias, inconsistency, indirectness, imprecision, and publication bias. Additionally, studies may be upgraded for three factors: large effect sizes, potential confounders, and dose-response relationships. The final GRADE evaluation of the quality of the evidence was then classified as high, moderate, low, or very low, as generated by GRADE pro software (version 3.6). Any disagreements were resolved by consultation with a third reviewer (JZ).

### 2.5 Statistical analysis

Statistical heterogeneity of each outcome was evaluated using the Cochrane Q statistic (statistical significance set at *P* < 0.10) and quantified by the *I^2^* index. The interpretation of the *I^2^* index was classified as low, moderate, and high with *I^2^* values ranging from 25%, 50%, and 75% respectively (29). Given the inevitable clinical heterogeneity of the included studies and the recommended methods of the references, we employed the random-effects model regardless of the results of the heterogeneity test (30). Since the included studies used different kits to detect irisin, the SMD with a 95% CI was used to calculate the pooled results. Prior to the meta-analysis of the correlation coefficients, we transformed Spearman’s correlation coefficient into Pearson’s correlation coefficient for unification, then meta-analysis were conducted using the inverse of the variance method and Fisher’s z-transformation with the methodologies described in the previous literature (31, 32).

The subgroup analysis and uni-variate meta-regression were conducted to determine the source of heterogeneity and to investigate associations between the effect size and several pre-defined related clinical and methodological factors. These included different age groups (≥ 60 vs. < 60 years), whether matched for age (matched vs. not matched), BMI (non-obese vs. obese), ethnicity (Asian vs. Caucasian), definition of the non-OP group (T > -1 vs. T < -1), publication language (English vs. Chinese), and ELISA kit (Phoenix vs. others).

Sensitivity analysis using the leave-one-out method was performed to assess the influence of each study on the pooled results and to explore the robustness of the meta-analysis. The results were considered robust if the findings remained unchanged after the exclusion of a single study, otherwise, the robustness was poor, and the findings needed to be treated with caution. Moreover, funnel plots, Begg’s test, and Egger’s test were examined to explore potential publication bias (33). We hypothesized no obvious publication bias existed if *P* > 0.05, otherwise significant publication bias existed. The impact of possible missing studies on the overall results was assessed using a nonparametric trim-and-fill method to judge the stability of the results. Consistency of results before and after applying the trim-and-fill method indicated robust findings (34).

A two-tailed *P* value < 0.1 indicated significance for subgroup comparisons and meta-regression analysis based on the tutorial (35, 36). A two-tailed *P* value < 0.05 was considered statistically significant for the test of overall effect. The meta-analysis and subgroup analysis were performed using RevMan software (Version 5.4, Nordic Cochrane Centre, Copenhagen, Denmark). Analyses of publication bias and meta-regression were conducted using the STATA software (Version 16; Stata Corporation, College Station, TX).

## 3 Results

### 3.1 Search results

**Figure 1:**
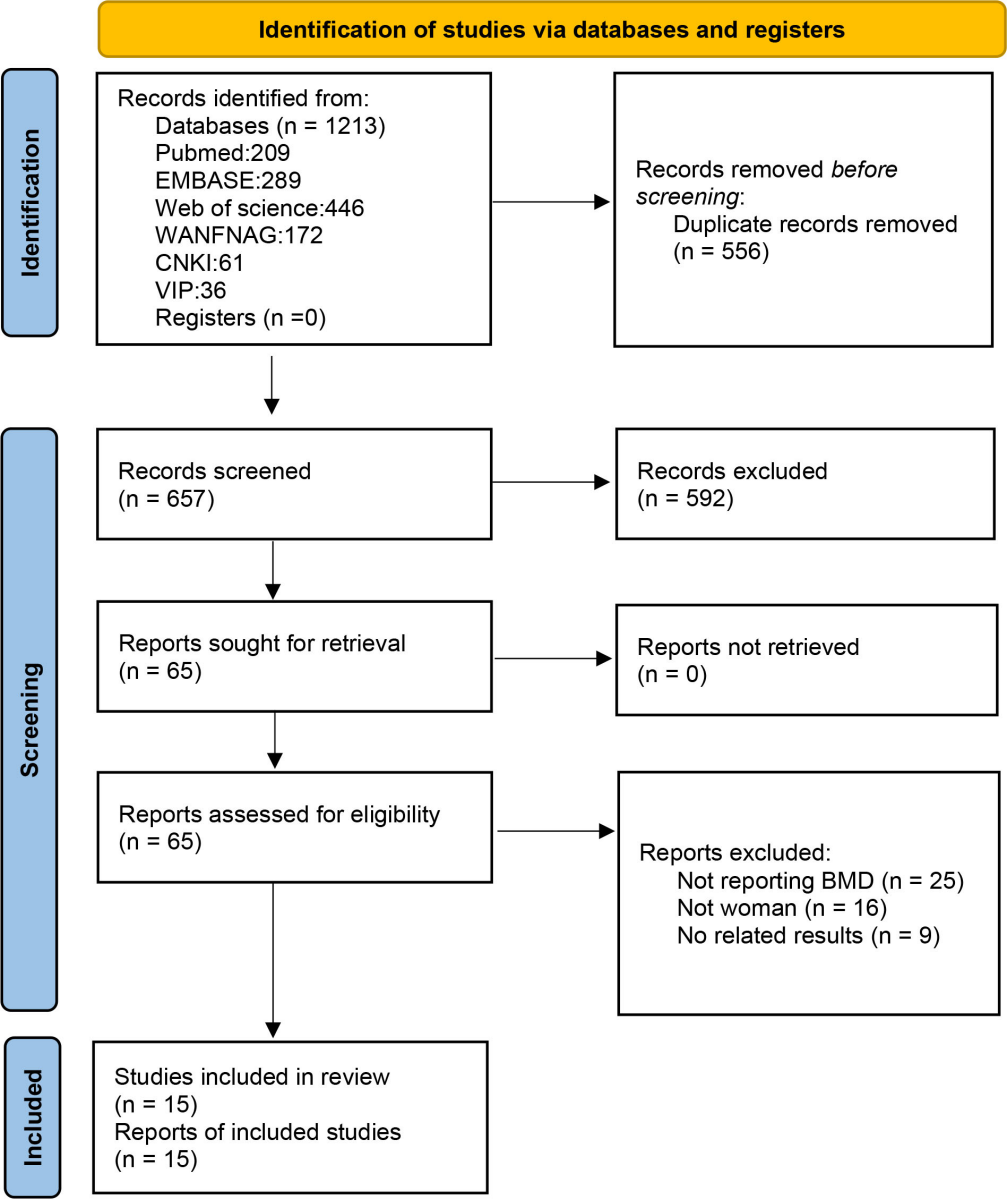
Flow diagram of the search process according to PRISMA.

A summary of the review process is presented in the flow chart in **Figure 1**. The initial search identified 1213 publications. After removing duplications, 657 publications remained. By carefully scanning titles and abstracts, we removed 592 irrelevant records and screened the full texts of remained 65 articles. Articles were excluded for the following reasons: 25 articles did not report BMD; 16 articles did not meet the inclusion criteria of population; and 9 articles did not report relevant outcomes. Finally,15 eligible studies that met our criteria were included in the systematic review and meta-analysis.

### 3.2 Study characteristics

The characteristics of the included studies are shown in **Table 1** (12–14, 18, 20, 22, 37–45). A total of 15 studies with 2856 participants were included in this systematic review and meta-analysis. These studies were published between 2014 and 2022. Nine studies were published in English, six were in Chinese. The studies originated from China (8 studies), Greece (2 studies), France (1 study), Italy (1 study), Iraq (1 study), Korea (1 study), and Turkey (1 study), including Asian and European populations. The age range of participants was from 18.5 ± 4.2 to 80.7 ± 1.7 years old. Most studies used DXA to measure BMD, except one study used Quantitative Computed Tomography (QCT) (18). However, the diagnostic criteria for OP varied across different studies as presented in **Table 1**. Specifically, for the outcome of irisin levels in the osteoporosis and control groups, 3/11 articles did not provide diagnostic criteria, and 7/11 articles used a diagnostic criterion of T score less than -2.5, while 1 article used a criterion of T score less than -2.0. Most studies detected irisin levels in serum, with only one measuring irisin in plasma (40). Furthermore, most of the studies measured irisin by commercial enzyme-linked immunosorbent assay (ELISA) kits from seven different corporations, though one study did not mention the ELISA kit manufacturer (38) and another did not report the measurement method (37). We did not exclude the literature including postmenopausal women who had previously received anti-osteoporosis treatment, because patients with severe osteoporosis or fractures generally require such therapy, and prior literature indicates that three months of teriparatide or denosumab treatment does not affect irisin levels (45).

**Table 1:** Basic characteristics of included studies.

First author	Year	Country	Study design	Participants	Definition of case	Definition of control	Number of Participants	Mean Age (years) (Mean ± SD)		BMI (kg/m^2^) (Mean ± SD)		ELISA kit manufacturer	Outcomes
							(case/control)	case	control	case	control		
Maïmoun (22)	2022	France	case-control study	Adolescents and young women	NA	NA	42	18.5 ± 4.2		21.8 ± 2.5		Biovendor, Czech Republic	③
Zhu (37)	2022	China	cross-sectional study	Postmenopausal	NA	NA	370/321	57.38 ± 11.27	55.36 ± 14.76	26.17 ± 4.65	25.31 ± 3.35	NA	①③④
Zhou (38)	2022	China	cross-sectional study	Postmenopausal	≤-2.5	>-1	90/50	58.98 ± 8.27	59.76 ± 8.13	23.21 ± 2.76	22.89 ± 2.83	ELISA, NA	①③
Liu (13)	2021	China	cross-sectional study	Postmenopausal with and without hip fracture	<-2.5	>-1.5	215/215	68.7 ± 11.7		23.7(3.2)	23.9(3.4)	Aviscera Biosciences, USA	①②③④
Roomi (12)	2021	Iraqi	cross-sectional study	Postmenopausal	<-2.5	>-1	80/95	59.73 ± 3.44	58.41 ± 3.51	NA	NA	Demeditec Diagnostic Testing GmbH, Germany	①③
Anastasilakis (20)	2021	Greece	case-control study	Postmenopausal	postmenopausal women with a low trauma hip fracture	postmenopausal women with knee or hip osteoarthritis	32/37	80.7 ± 1.7	69.6 ± 1.2	27.2 ± 1.1	32.3 ± 1.1	EK-067-29, Phoenix Pharmaceuticals, USA	②
Zhang (39)	2020	China	cross-sectional study	Postmenopausal patients with T2MD	≤-2.5	≥-1	50/50	52. 8 ± 1.4	52. 0 ± 1.2	23.71 ± 3.05	24.15 ± 2.80	Huiying Biotechnology, China	①③④
Shi (40)	2019	China	cross-sectional study	Postmenopausal	NA	NA	EARLY^a^ 21/47; LATE^b^ 39/51	EARLY^a^ 58.48 ± 2.98; LATE^b^ 67.60 ± 3.93	EARLY^a^ 54.88 ± 2.59; LATE^b^ 62.30 ± 3.95	NA	NA	EK-086-43, Phoenix Pharmaceuticals, USA	①③
Duan (41)	2019	China	cross-sectional study	Postmenopausal patients with T2MD	NA	NA	40/24	68.2(52∼82)		NA	NA	USCN KIT INC., China	①③
Park (18)	2019	Korea	cross-sectional study	Postmenopausal	NA	NA	153	72.20 ± 5.96		23.38 ± 3.45		EK-067-29, Phoenix Pharmaceuticals, USA	②④
Yan (14)	2018	China	cross-sectional, case-control study	Postmenopausal with or without minimal trauma hip fractures	women with minimal trauma hip fractures	women without fracture	160/160	78 (73–80)	76 (72–79)	20.3 (18.8–22.2)	23.4 (21.9–26.0)	Phoenix Pharmaceuticals, USA	②③④
Liang (42)	2017	China	cross-sectional study	Postmenopausal women with T2MD	<-2.5	>-1	91/50	66 ± 8	58 ± 7	23.3 ± 3.2	26.3 ± 2.9	Cusabio, China	①③④
Engin-Üstün (43)	2016	Turkey	cross-sectional study	Postmenopausal	<-2.5	>-1	88/88	55 (41-82)	56 (43-81)	31.0 ± 4.4	31.4 ± 4.8	Biovendor, Czech Republic	①
Palermo (44)	2015	Italy	cross-sectional study	Postmenopausal	<-2.5	>-2.5	36/36	65.6 ± 6.7	62.9 ± 5.1	25.7 ± 2.8	26.6 ± 3.0	AG-45A-0046EK-KI01, Adipogen, Switzerland	①②③④
Anastasilakis (45)	2014	Greece	cross-sectional study	Postmenopausal	≤-2.0	>-2	75/50	65.6 ± 1.5	64.6 ± 2.3	29.6 ± 1.1	31.2 ± 1.3	Phoenix Pharmaceuticals, USA	①②

BMI, body mass index; ELISA, enzyme-linked immunosorbent assay; T2MD, type 2 diabetes mellitus; NA, not available.

a: Early postmenopausal group (within 10 years after menopause); b: Late postmenopausal group (more than 10 years after menopause).

Outcomes: ①Irisin levels in postmenopausal women with or without osteoporosis; ②Irisin levels in postmenopausal women with or without fractures; ③Correlation coefficient between serum/plasma irisin levels and bone mineral density. ④Correlation coefficient between serum/plasma irisin levels and age.

### 3.3 Methodological quality

The NOS scale was used to assess the methodological quality of the included articles. Detailed scores for each study are shown in **Supplementary Table 1**. One study scored 5, two scored 6, seven studies scored 7, four scored 8, and one scored 9. Overall, the methodological quality of the included studies was moderate to high. Three studies of moderate quality, scoring 5–6, exhibited specific deficiencies: three lacked a definition of the case group, resulting in an adequate case definition score of 0 (37, 40, 41); two included hospital population controls, which led to a selection of controls score of 0 (40, 41); one did not provide the definition of the control group, resulting in a score of 0 for the definition of controls (41); three controlled for various confounders but not for age, resulting in the comparability section score of 1 (37, 40, 41); One did not report the detection kit used, thus resulting in an ascertainment of exposure score of 0 (37).

### 3.4 Meta-analysis of irisin levels in PMOP individuals

**Figure 2:**
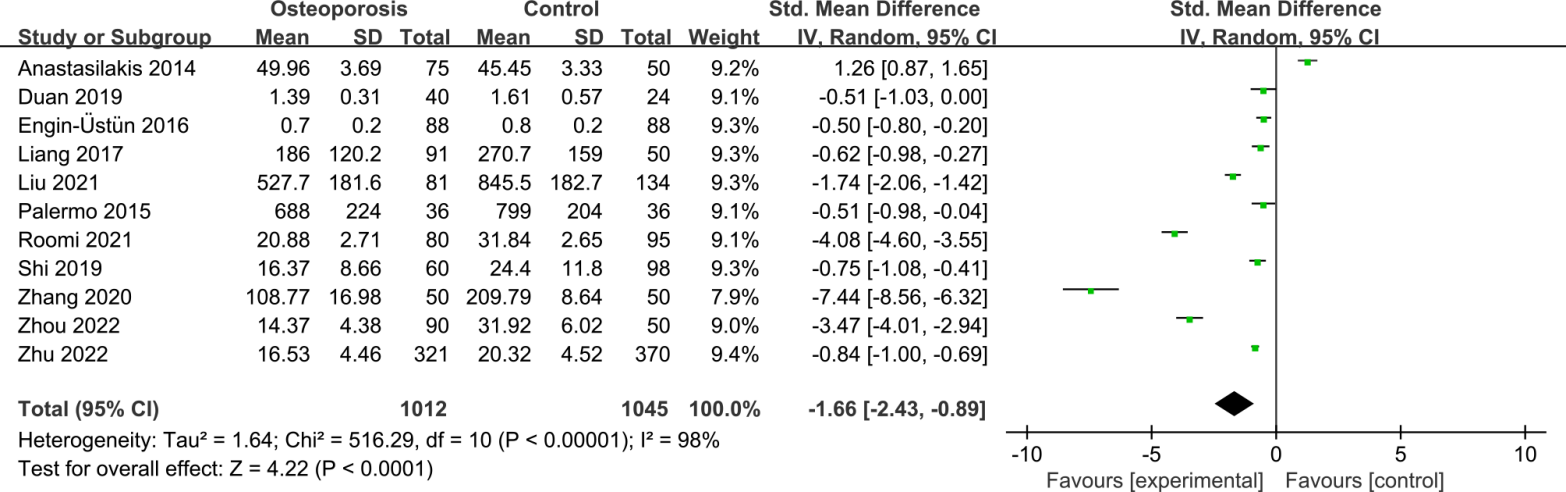
Forest plot of differences in irisin levels between postmenopausal women with osteoporosis and with normal bone mineral density.

11 studies reported irisin levels in 1012 PMOP cases and 1045 non-PMOP controls. The pooled result suggested that individuals with PMOP had significantly lower circulating irisin levels than non-PMOP controls (SMD = -1.66, 95% CI: -2.43 to -0.89, *P* < 0.0001; *I^2^* = 98%, *P* < 0.00001) (**Figure 2**).

### 3.5 Meta-analysis of irisin levels in postmenopausal women with fractures

**Figure 3:**
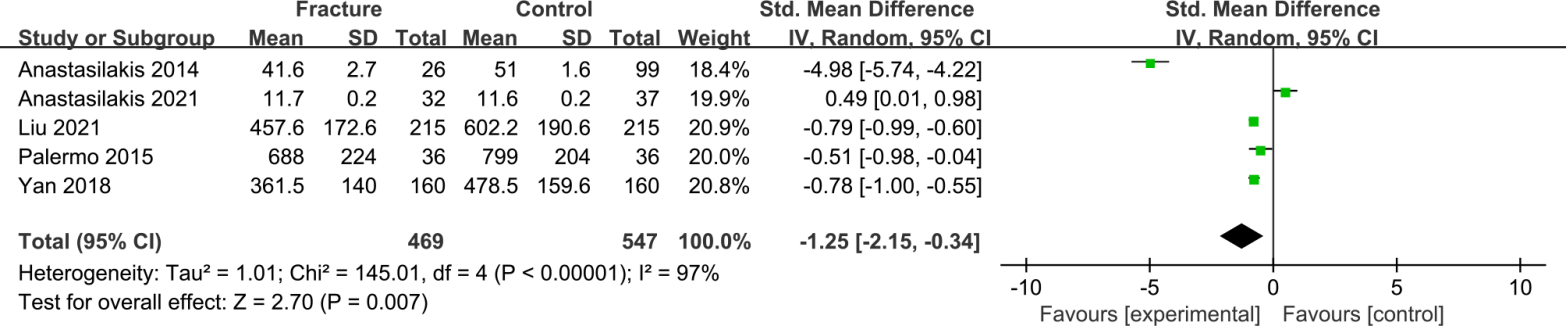
Forest plot of differences in irisin levels in postmenopausal women with osteoporotic fractures and without fractures.

The forest plot including 5 studies with 469 fracture cases and 547 non-fracture controls showed that postmenopausal women with fractures had significantly lower irisin levels than non-fracture controls (SMD = -1.25, 95% CI: -2.15 to -0.34, *P* = 0.007; *I^2^* = 97%, *P* < 0.00001) (**Figure 3**).

### 3.6 Meta-analysis of the correlation between irisin levels and the BMD

**Figure 4:**
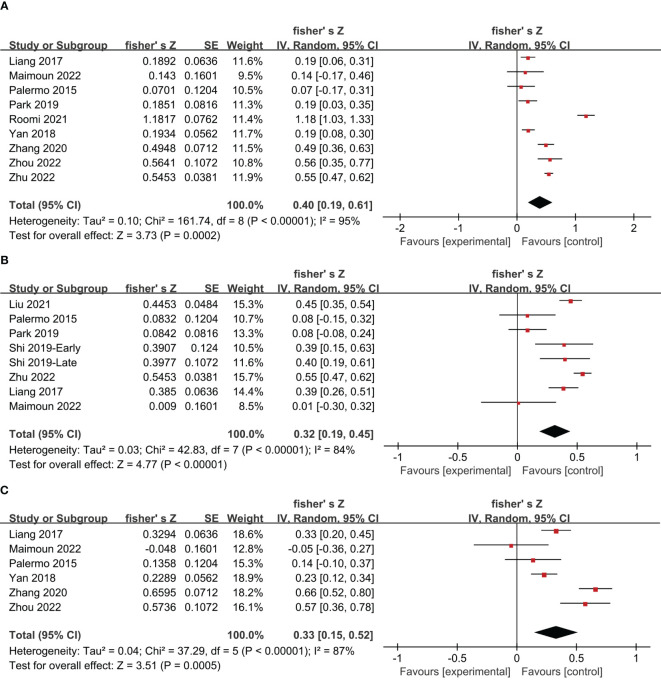
Forest plot of meta-analysis of the correlation coefficient between irisin and bone mineral density in the lumbar **(A)**, femoral **(B)**, and femoral neck **(C)**.

11 studies on postmenopausal women and 1 study on young women reported Pearson’s or Spearman’s correlation coefficients between irisin levels and BMD. Using Fisher’s Z transform, we performed a pooled analysis of the correlations between irisin levels and BMD. The meta-analysis generated a summary Fisher’s Z value of 0.40 (95% CI: 0.19 to 0.61, *P* = 0.0002; *I^2^* = 95%, *P* < 0.00001), 0.32 (95% CI: 0.19 to 0.45, *P* < 0.00001; *I^2^* = 84%, *P* < 0.00001), and 0.33 (95% CI: 0.15 to 0.52, *P* = 0.0005; *I^2^* = 87%, *P* < 0.00001) for lumbar, femoral and femoral neck, respectively (**Figure 4**). Therefore, a summary r value was 0.37 (95% CI: 0.18 to 0.54), 0.30 (95% CI: 0.18 to 0.42), and 0.31 (95% CI: 0.14 to 0.47) for lumbar, femoral, and femoral neck, respectively. This meta-analysis of correlation coefficients revealed a significantly positive relationship between irisin levels and BMD for women.

### 3.7 Subgroup analysis of irisin levels in PMOP individuals

Due to the significant heterogeneity of the included studies, subgroup analysis were conducted based on different ages, whether matched for age, BMI, ethnicity, definition of non-OP group, publication language, and ELISA kit to explore potential influencing factors. As evidenced in **Table 2**, no single factor emerged as a definitive source of heterogeneity by subgroup analysis, and statistical heterogeneity remained high across subgroups. It is worth noting that some subgroup results have changed, losing statistical significance, or showing differences between subgroups as detailed below.

**Table 2:** Subgroup analysis and meta-regression of irisin levels in PMOP individuals.

Subgroup analysis		Number of included studies	Number of cases	Number of controls	Heterogeneity		Random-effects model meta-analysis results		Differences between subgroups		Meta-regression	
					*I^2^*	*P* value	SMD (95% CI)	*P* value	*I^2^*	*P* value	Adjusted *R^2^*	*P* value
Age	≥60	6	393	392	96%	<0.00001	-0.48 [-1.30, 0.34]	0.25	89.1%	0.002	30.16%	0.050
	<60	5	629	653	99%	<0.00001	-3.18 [-4.72, -1.64]	<0.0001				
Age match	Matched	6	420	408	99%	<0.00001	-1.99 [-3.49, -0.50]	0.009	0	0.46	-12.23%	0.789
	Not matched	5	592	637	97%	<0.00001	-1.34 [-2.23, -0.45]	0.003				
BMI	Non-obese	7	729	788	98%	<0.00001	-2.04 [-2.87, -1.21]	<0.00001	83.6%	0.01	9.71%	0.221
	Obese	2	163	138	98%	<0.00001	0.38 [-1.35, 2.10]	0.67				
Ethnicity	Asian	7	733	776	97%	<0.00001	-2.04 [-2.88, -1.20]	<0.0001	7.4%	0.30	-4.25%	0.445
	Caucasian	4	279	269	99%	<0.00001	-0.95 [-2.83, 0.94]	0.32				
Control group	T>-1	5	399	333	99%	<0.00001	-3.16 [-5.00, -1.33]	0.0007	78%	0.03	17.90%	0.166
	T<-1	3	192	220	99%	<0.00001	-0.33 [-2.18, 1.52]	0.73				
Language	English	5	360	403	99%	<0.00001	-1.11 [-2.58, 0.36]	0.14	20.5%	0.26	-4.93%	0.463
	Chinese	6	652	642	98%	<0.00001	-2.12 [-3.12, -1.12]	<0.0001				
ELISA kit	Phoenix	2	135	148	98%	<0.00001	0.26 [-1.71, 2.22]	0.80	78.4%	0.03	13.77%	0.148
	others	9	877	897	98%	<0.00001	-2.09 [-2.92, -1.26]	<0.00001				

#### 3.7.1 Age

Subgroup analysis based on age (cutoff at 60 years) revealed contrasting findings. Six studies focusing on individuals over 60 showed no significant difference in irisin levels between PMOP and non-PMOP controls (SMD = -0.48, 95% CI: -1.30 to 0.34, *P* = 0.25; *I^2^* = 96%, *P* < 0.00001). Conversely, five studies involving participants below 60 years found significantly lower irisin levels in the PMOP group (SMD = -3.18, 95% CI: -4.72 to -1.64, *P* < 0.0001; *I^2^* = 99%, *P* < 0.00001), with a notable difference between these age subgroups (*P* = 0.002).

#### 3.7.2 Matching for age

In studies in which age was matched between OP cases and non-OP controls, significantly decreased irisin levels were observed in the PMOP group (SMD = -1.99, 95% CI: -3.49 to -0.50, *P* = 0.009; *I^2^* = 99%, *P* < 0.00001). Similarly, studies without age matching also reported lower irisin levels in the PMOP group (SMD = -1.34, 95% CI: -2.23 to -0.45, *P* = 0.003; *I^2^* = 97%, *P* < 0.00001). The results of the two subgroups were not statistically different (*P* = 0.46).

#### 3.7.3 Body mass index

Because specific BMI values reflect a higher percentage of body fat in some Asian populations than in white or European populations, we used a combination of WHO obesity criteria and WHO-recommended BMI cutoff points for Asians when considering public health actions (46). Participants were categorized into obese (≥ 30 kg/m^2^ in Caucasian, ≥ 27.5 kg/m^2^ in Asian) and non-obese (< 30 kg/m^2^ in Caucasian, < 27.5 kg/m^2^ in Asian) subgroups. Studies not reporting the BMI values were excluded. As a result, in the non-obese subgroup, seven studies indicated lower irisin levels in PMOP women than in non-PMOP controls (SMD = -2.04, 95% CI: -2.87 to -1.21, *P* < 0.00001; *I^2^* = 98%, *P* < 0.00001). In contrast, in the obese subgroup, two studies showed no significant difference in irisin levels (SMD = 0.38, 95% CI: -1.35 to 2.10, *P* = 0.67; *I^2^* = 98%, *P* < 0.00001). A significant disparity was observed between the obese and non-obese subgroups (*P* = 0.01).

#### 3.7.4 Ethnicity

An ethnic comparison revealed that seven studies on Asian participants found significantly lower irisin levels in PMOP women than in non-PMOP controls (SMD = -2.04, 95% CI: -2.88 to -1.20, *P* < 0.0001; *I^2^* = 97%, *P* < 0.00001). However, such a difference was not found in four studies on Caucasian subjects (SMD = -0.95, 95% CI: -2.83 to 0.94, *P* = 0.32; *I^2^* = 99%, *P* < 0.00001). No substantial variation was found between these ethnic subgroups (*P* = 0.30).

#### 3.7.5 Definition of non-OP group

Divergent definitions of non-OP controls influenced the outcomes. Among the studies included, eight studies defined osteoporosis as a T-score was less than -2.5 based on WHO criteria. Five studies defining non-OP control as a T-score greater than -1 reported significantly lower irisin levels in PMOP than in non-PMOP control (SMD = -3.16, 95% CI: -5.00 to -1.33, *P* = 0.0007; *I^2^* = 99%, *P* < 0.00001). In contrast, three studies defining non-OP control as a T score less than -1 found no significant difference (SMD = -0.33, 95% CI: -2.18 to 1.52, *P* = 0.73; *I^2^* = 99%, *P* < 0.00001). It is worth noting that there was a significant difference between the subgroups (*P* = 0.03).

#### 3.7.6 Language

Irisin levels in PMOP participants were significantly lower in PMOP women than in non-PMOP controls in Chinese-language studies (SMD = -2.12, 95% CI: -3.12 to -1.12, *P* < 0.0001; *I^2^=* 98%, *P* < 0.00001). Conversely, English-language studies showed no significant difference between the PMOP group and non-PMOP controls (SMD = -1.11, 95% CI: -2.58 to 0.36, *P =* 0.14; *I^2^* = 99%, *P* < 0.00001). No marked difference was found between these language-based subgroups (*P* = 0.26).

#### 3.7.7 ELISA kit

Analysis of two studies using ELISA kits from Phoenix company revealed no significant difference in irisin levels between the PMOP group and non-PMOP controls (SMD = 0.26, 95% CI: -1.71 to 2.22, *P =* 0.80; *I^2^* = 98%, *P* < 0.00001). However, pooled results of nine studies using kits from other companies showed lower irisin levels in the PMOP group than in non-PMOP controls (SMD = -2.09, 95% CI: -2.92 to -1.26, *P* < 0.00001; *I^2^* = 98%, *P* < 0.00001), indicating significant differences between these kit subgroups (*P* = 0.03).

### 3.8 Subgroup analysis of irisin levels in postmenopausal women with osteoporotic fractures

The five included studies were published in English articles and involved participants above 60 years of age. Thus, subgroup analysis based on age and language were not feasible. Therefore, we conducted further subgroup analysis focusing on age matching, BMI, ethnicity, and the type of ELISA kit used, with detailed findings presented in **Table 3**.

**Table 3:** Subgroup analysis and meta-regression of irisin levels in postmenopausal women with osteoporotic fractures.

Subgroup analysis		Number of included studies	No. of cases	No. of control	Heterogeneity		Random-effects model meta-analysis results		Differences between subgroups		Meta-regression	
					*I^2^*	*P* value	SMD (95% CI)	*P* value	*I^2^*	*P* value	Adjusted *R^2^*	*P* value
Age match	Matched	4	437	510	97%	<0.00001	-1.67 [-2.66, -0.69]	0.0008	93.4	0.0001	-3.26%	0.417
	Not match	1	32	37	NA	NA	0.49 [0.01, 0.98]	0.04				
BMI	Non-obese	3	411	411	0%	0.55	-0.76 [-0.90, -0.62]	<0.00001	0%	0.59	-13.35%	0.509
	Obese	2	58	136	99%	<0.00001	-2.23 [-7.60, 3.13]	0.41				
Ethnicity	Asian	2	375	375	0%	0.92	-0.79 [-0.94, -0.64]	<0.00001	0%	0.53	-28.06%	0.715
	Caucasian	3	94	172	99%	<0.00001	-1.65 [-4.36, 1.06]	0.23				
ELISA Kit	Phoenix	3	218	296	99%	<0.00001	-1.72 [-3.94, 0.49]	0.13	0%	0.38	-24.18%	0.647
	others	2	251	251	15%	0.28	-0.74 [-0.96, -0.52]	<0.00001				

NA, not applicable.

#### 3.8.1 Matching for age

Four studies with age matching between fracture cases and non-fracture controls revealed decreased irisin levels in postmenopausal women with fractures (SMD = -1.67, 95% CI: -2.66 to -0.69, *P* < 0.0008; *I^2^* = 97%, *P* < 0.00001). Contrastingly, one study without age matching indicated elevated irisin levels in fracture cases than controls (SMD = 0.49, 95% CI: 0.01 to 0.98, *P* = 0.04; *I^2^* = NA, *P =* NA), highlighting a significant disparity between these subgroups (*P* = 0.0001).

#### 3.8.2 Body mass index

Three articles provided data on irisin levels in non-obese individuals. The pooled results showed that, in normal-weight individuals, the irisin concentration was significantly lower in fracture cases than in non-fracture controls (SMD = -0.76, 95% CI: -0.90 to -0.62, *P* < 0.00001; *I^2^* = 99%, *P* < 0.00001). Meanwhile, in the two studies assessing obese postmenopausal women, the difference in irisin levels between those with fractures and controls was not statistically significant (SMD = -2.23, 95% CI: -7.60 to 3.13, *P* = 0.41; *I^2^* = 99%, *P* < 0.00001), with no discernible differences between these subgroups (*P* = 0.59).

#### 3.8.3 Ethnicity

Our findings indicated lower irisin levels in postmenopausal Asian women with fractures than in non-fracture controls (SMD = -0.79, 95% CI: -0.94 to -0.64, *P* < 0.00001; *I^2^* = 0%, *P* = 0.92). However, in Caucasian populations, the difference was not significant (SMD = -1.65, 95% CI: -4.36 to 1.06, *P =* 0.23; *I^2^* = 99%, *P* < 0.00001). There were no significant differences observed among the diverse ethnic subgroups (*P* = 0.53).

#### 3.8.4 ELISA kit

In the subgroup analysis of three studies using Phoenix ELISA kits and two using kits from other companies, we noted that irisin levels were lower in postmenopausal women with fractures than non-fracture controls in the studies utilizing non-Phoenix kits (SMD = -0.74, 95% CI: -0.96 to -0.52, *P* < 0.00001; *I^2^* = 15%, *P* = 0.28). Conversely, studies using Phoenix kits did not show a significant difference (SMD = -1.72, 95% CI: -3.94 to 0.49, *P =* 0.13; *I^2^* = 99%, *P* < 0.00001). No significant differences were identified among subgroups based on the type of ELISA kit used (*P* = 0.38).

### 3.9 Subgroup analysis of the correlation between irisin levels and the BMD

We conducted subgroup analysis of the correlation between irisin levels and the BMD across various factors such as age, ethnicity, publication language, ELISA kit, and menopause status as shown in **Table 4**.

**Table 4:** (A) Subgroup analysis and meta-regression of the correlation of irisin levels and lumbar BMD.

Subgroup analysis		Number of included studies	Heterogeneity		Random-effects model meta-analysis results		Differences between subgroups		Meta-regression	
			*I^2^*	*P* value	Fisher’s Z (95% CI)	*P* value	*I^2^*	*P* value	Adjusted *R^2^*	*P* value
Age	≥60	4	0%	0.82	0.18 [0.11, 0.25]	<0.00001	87.8%	0.004	41.99%	0.049
	<60	5	94%	<0.00001	0.60 [0.32, 0.88]	<0.0001				
Ethnicity	Asian	6	90%	<0.00001	0.36 [0.20, 0.52]	<0.0001	0%	0.79	-9.48%	0.643
	Caucasian	3	97%	<0.00001	0.47 [-0.34, 1.29]	0.26				
Language	English	5	97%	<0.00001	0.36 [-0.09, 0.80]	0.11	0%	0.73	-13.72%	0.747
	Chinese	4	88%	<0.0001	0.44 [0.26, 0.62]	<0.00001				
ELISA kit	Phoenix	2	0%	0.93	0.19 [0.10, 0.28]	<0.0001	75.1%	0.04	0.62%	0.355
	others	7	95%	<0.00001	0.47 [0.21, 0.72]	0.0003				
Menopause	postmenopause	8	96%	<0.00001	0.43 [0.21, 0.65]	0.0002	53.1%	0.14	-6.16%	0.502
	premenopause	1	NA	NA	0.14 [-0.17, 0.46]	0.37				

NA, not applicable.

**Table 4:** (B) Subgroup analysis and meta-regression of the correlation of irisin levels and femoral BMD.

Subgroup analysis		Number of included studies	Heterogeneity		Random-effects model meta-analysis results		Differences between subgroups		Meta-regression	
			*I^2^*	*P* value	Fisher’s Z (95% CI)	*P* value	*I^2^*	*P* value	Adjusted *R^2^*	*P* value
Age	≥60	5	75%	0.001	0.31 [0.17, 0.44]	<0.00001	0%	0.98	-17.07%	0.822
	<60	2	91%	0.001	0.30 [-0.22, 0.82]	0.26				
Ethnicity	Asian	5	82%	<0.0001	0.38 [0.26, 0.51]	<0.00001	87.5%	0.005	41.00%	0.068
	Caucasian	2	0%	0.71	0.06 [-0.13, 0.24]	0.56				
Language	English	4	87%	<0.0001	0.17 [-0.07, 0.42]	0.17	76.2%	0.04	33.88%	0.083
	Chinese	3	52%	0.10	0.45 [0.35, 0.56]	<0.00001				
ELISA kit	Phoenix	2	73%	0.03	0.28 [0.06, 0.50]	0.01	0%	0.62	-13.93%	0.750
	others	5	83%	<0.0001	0.35 [0.20, 0.49]	<0.00001				
Menopause	postmenopause	6	83%	<0.00001	0.35 [0.22, 0.48]	<0.00001	73.8%	0.05	14.81%	0.202
	premenopause	1	NA	NA	0.01 [-0.30, 0.32]	0.96				

NA, not applicable.

**Table 4:** (C) Subgroup analysis and meta-regression of the correlation of irisin levels and femoral neck BMD.

Subgroup analysis		Number of included studies	Heterogeneity		Random-effects model meta-analysis results		Differences between subgroups		Meta-regression	
			*I^2^*	*P* value	Fisher’s Z (95% CI)	*P* value	*I^2^*	*P* value	Adjusted *R^2^*	*P* value
Age	≥60	3	22%	0.28	0.26 [0.16, 0.35]	<0.00001	0%	0.37	5.15%	0.414
	<60	3	88%	0.0003	0.42 [0.07, 0.77]	0.02				
Ethnicity	Asian	4	89%	<0.00001	0.44 [0.23, 0.65]	<0.0001	85.2%	0.009	42.85%	0.107
	Caucasian	2	0%	0.36	0.07 [-0.12, 0.26]	0.47				
Language	English	3	31%	0.24	0.16 [0.02, 0.30]	0.02	85.8%	0.008	65.44%	0.051
	Chinese	3	84%	0.02	0.52 [0.29, 0.74]	<0.00001				
ELISA kit	Phoenix	1	NA	NA	0.23 [0.12, 0.34]	<0.0001	0%	0.34	-26.61%	0.713
	others	5	86%	<0.00001	0.35 [0.13, 0.58]	0.002				
Menopause	postmenopause	5	87%	<0.00001	0.39 [0.20, 0.58]	<0.0001	81.7%	0.02	24.85%	0.192
	premenopause	1	NA	NA	-0.05 [-0.36, 0.27]	0.76				

NA, not applicable.

#### 3.9.1 Age

Subgroup analysis based on age demonstrated that, in the subgroup of participants below 60, the correlation between irisin and femoral BMD was not significant (Fisher’s Z = 0.30, 95% CI: -0.22 to 0.82, *P* = 0.26; *I^2^* = 91%, *P* = 0.001). However, significant correlations were observed in other age groups and at different bone sites, as detailed in **Table 4**.

#### 3.9.2 Ethnicity

The correlation between irisin levels and BMD varied according to ethnicity. The observed correlation between irisin levels and BMD in women was consistent in Asia, but inconsistent for the studies performed in Caucasian populations at the lumbar, femoral, and femoral neck detailed in **Table 4**.

#### 3.9.3 Language

The correlation between irisin and BMD also varied across studies published in different languages. In English-language publications, irisin was not correlated with BMD for lumbar, and femoral, but was correlated with femoral neck BMD. In contrast, Chinese-language studies indicated a positive correlation between irisin and BMD at all measured sites as shown in **Table 4**.

#### 3.9.4 ELISA kit

Subgroups based on the type of ELISA kit used, whether a Phoenix or non-Phoenix brand, consistently showed a correlation between irisin and BMD at the lumbar, femoral, and femoral neck sites.

#### 3.9.5 Menopause status

When the comparison was stratified by menopause status, in the postmenopausal group, irisin level were positively correlated with BMD for the lumbar, femoral, and femoral neck. However, irisin levels were not correlated with BMD in the premenopausal group, with significant differences between premenopausal and postmenopausal subgroups at the femoral (*P* < 0.05) and femoral neck (*P* < 0.02) sites.

### 3.10 Meta-regression analysis

Given the high level of heterogeneity in our meta-analysis (*I^2^* > 50%), we explored the possible sources of heterogeneity using meta-regression. The meta-regression models identified the age separated by 60 years (*R²* = 30.16%, *P* = 0.050) as a significant moderator in the relationship between irisin levels and OP (**Table 2**). Other potential confounding factors, including age matching for cases and controls, obesity status, ethnicity, control group definition, the language of publication, and ELISA kit type, did not significantly influence the association between irisin levels and OP (all *P* values > 0.1, **Table 2**). In the context of the relationship between irisin and fractures, meta-regression analysis indicated that factors such as age matching, BMI, ethnicity, and ELISA kit type did not contribute to the observed heterogeneity (**Table 3**). Meta-regression of the correlation of irisin levels and BMD showed that age was a significant moderator (*R²* = 41.99%, *P* = 0.049) for the correlation of irisin levels and lumbar BMD. Additionally, both ethnicity (*R*² = 41.00%, *P* = 0.068) and language (*R²* = 33.88%, *P* = 0.083) were significant moderators for the correlation between irisin levels and femoral BMD. Furthermore, publication language also played a significant moderating role in the correlation between irisin levels and femoral neck BMD (*R²* = 65.44%, *P* = 0.051), as shown in **Table 4**.

### 3.11 Assessment of publication bias and sensitivity analysis

A funnel plot was drawn to evaluate the publication bias of studies investigating the association between irisin levels and PMOP. The funnel plot exhibited a symmetrical shape (**Supplementary Figure 1**). The results of the Begg’s test (*P* = 0.087) and the Egger’s test (*P* = 0.176) showed no statistical significance. The trim and fill results suggested that one study was missing. After imputing the missing study, the mean overall effect size was SMD = -1.99, 95% CI: -2.94 to -1.04, *P* = 0.000. Taken together, these analyses suggested that there was no publication bias among included studies in the analysis of irisin levels in postmenopausal women with OP. For the outcome of irisin levels in postmenopausal women with fracture, the results of Begg’s test (*P* = 0.806) and Egger’s test (*P* = 0.558) indicated no publication bias. Similarly, no significant publication bias was found for the correlation between irisin and lumbar BMD (Begg’s test *P* = 0.754, Egger’s test *P* = 0.606), or femoral neck BMD (Begg’s test *P* = 1.0, Egger’s test *P* = 0.756). However, significant publication bias was detected in studies examining the correlation between irisin and femoral BMD (Begg’s test *P* = 0.174, Egger’s test *P* = 0.023).

Results of sensitivity analysis confirmed the robustness of the pooled result that PMOP had significantly lower irisin levels than controls (**Supplementary Table 2A**). For postmenopausal women with fractures, the sensitivity analysis revealed a notable alteration when omitting Liu, Yan, and Anastasilakis’ study at one time (**Supplementary Table 2B**), suggesting that the pooled test could be influenced by a single study. Therefore, the conclusion should be interpreted carefully and further tested. Sensitivity analysis of the correlation between irisin levels and the BMD confirmed the robustness of the pooled results (**Supplementary Table 2C**).

### 3.12 Assessment of evidence quality

The quality of the evidence was assessed using the GRADE framework. The evidence for decreased irisin levels in PMOP was rated as “very low.” Similarly, the quality of evidence for decreased irisin levels in women with fractures was also rated as “very low.” Furthermore, the association between irisin and BMD was supported by “very-low” quality evidence. The most common reason for downgrading the quality of each outcome was inconsistency due to high heterogeneity (*I^2^* > 50%). In addition, the quality of evidence for the association between irisin and femoral neck BMD was downgraded due to reporting bias, as indicated by a *P* value of less than 0.05 for Egger’s test. A summary of the GRADE assessments of each outcome was shown in the **Supplementary File Table 3**.

## 4 Discussion

Osteoporosis is a common chronic disorder with high morbidity, significantly impacting the quality of life. Physical exercise is widely recognized for its beneficial effects on bone health, enhancing bone formation, and improving BMD through both direct mechanical forces and indirect neurohormonal mechanisms (47, 48). Among these mechanisms, muscle contractions during physical activity stimulate the production of irisin, a myokine that plays a crucial role in bone metabolism (49, 50). Irisin promotes osteoblast differentiation and activity while inhibiting osteoclastogenesis, which leads to increased bone formation and mineral density (51). This dual action establishes a biological link between muscle activity and improved bone strength. The upregulation of irisin in response to exercise highlights its therapeutic potential in the prevention and management of osteoporosis and other bone-related disorders.

However, the relationship between circulating irisin levels and OP/fractures remains conflict. To clarify this relationship, we conducted this meta-analysis and detailed subgroup analysis including 15 studies with a total of 2856 female participants evaluating circulating irisin levels. Our results revealed that postmenopausal women with OP exhibited significantly lower irisin levels than their non-PMOP counterparts. A similar trend was observed in postmenopausal women with a history of fractures. Notably, a positive correlation was identified between circulating irisin levels and BMD in postmenopausal women.

The outcomes of our meta-analysis supported the hypothesis that irisin could serve as a protective biomarker for bone health, especially in postmenopausal women. This was consistent with numerous experimental studies exploring irisin’s role in bone homeostasis. The potential beneficial effect of irisin on bone was based on the mechanism of its osteogenic function. Irisin has been shown to promote osteogenesis of bone marrow mesenchymal stem cells (52) and to enhance osteoblast precursors differentiation and proliferation through activating the p38 mitogen-activated protein kinase (MAPK)/extracellular signal-regulating kinase (ERK) pathway (53). Additionally, irisin inhibited RANKL-stimulated osteoclast activation by suppressing NF-kB signaling (54). Moreover, irisin regulates bone formation relative to the mechanisms of cell death, including apoptosis, autophagy, pyroptosis, and ferroptosis (55). Clinical studies across diverse populations, including rheumatoid arthritis patients and older adults, have further validated irisin’s regulatory role in bone remodeling (11, 56).

While our findings were roughly consistent with the previous Zhou’s meta-analysis (24), our study offered a more focused examination of postmenopausal and young women and was the first to analyze whether menopause status influenced the relationship between irisin and BMD. By performing subgroup analysis of potential confounding factors, we identified the specific circumstances under which irisin could be a suitable biomarker for OP. Additionally, our study included a larger sample size with more articles included, providing a greater level of detail. Furthermore, the use of SMD as the effect size eliminated differences in units of measurement across various kits used in different studies. Therefore, our analysis reinforced the data quality and reliability compared to the previous meta-analysis and was more specific for women.

Notably, our meta-analysis exhibited significant heterogeneity across outcomes. To address the heterogeneity, in addition to using the random effect model for meta-analysis, we also conducted multiple subgroup analysis and meta-regression. Meanwhile, the results of sensitivity analysis and publication bias analysis proved the robustness of our results. Importantly, our results revealed that in specific subgroups, such as in postmenopausal women under 60 years of age, regardless of whether cases and controls were age-matched, of non-obese, of Asian race, with a control group T score greater than -1, using non-Phoenix ELISA kit, irisin levels were consistently lower in postmenopausal individuals with OP than in non-OP controls. These findings suggested a potential application of irisin as a biomarker within these specific contexts.

The subgroup analysis did not identify any subgroup factors as a source of heterogeneity. However, meta-regression analysis revealed that age significantly moderated the relationship between irisin levels and OP as well as the correlation between irisin levels and lumbar BMD. Additionally, ethnicity and language appeared to influence the correlation between irisin levels and femoral BMD. Furthermore, language also had a remarkable influence on the correlation between irisin levels and femoral neck BMD. The results of the meta-regression analysis suggested that the above factors may be the source of heterogeneity. According to the Cochrane Handbook, it is possible that the effect of a characteristic may not always be identified using subgroup analysis or meta-regression due to the substantial variability in the characteristics of participants across studies, while subgroup analysis and meta-regression are limited to summarizing data at the level of the study. The heterogeneity might be attributed to other variations in clinical features of the study populations such as metabolic status, body composition, hormone levels, and lifestyle factors. However, due to the lack of data in the literature, we could not analyze these potential confounders. Future studies should aim to expand the sample size, enhance the representation of the population, and select reliable detection methods to address the heterogeneity.

Subgroup analysis and meta-regression were conducted with a 60-year age threshold, as menopause typically begins around age 51, and the International Menopause Society recommends hormone therapy for fracture prevention between ages 50–60 or within 10 years after menopause (57). Thus, early detection and treatment of osteoporosis before the age of 60 are particularly crucial. Our findings implied that irisin may be a sensitive marker of bone mass loss in the early menopause stage but not in the late menopause stage. The results of the meta-regression confirmed that age separated by sixty years was an influential factor in the relationship between irisin and osteoporosis. One possible explanation is that bone loss accelerates during the late stage of menopausal transition and continues for the next two decades (58). Consequently, early menopause at the age of 50–60 has a rapid and significant change in bone mass and is an essential period for the detection of bone loss and the prompt diagnosis of osteoporosis. Conversely, in the late postmenopausal stage (more than 10 years), age-related skeletal muscle loss is prevalent in the elderly (18). It is possible that aging and muscle atrophy may decrease irisin levels, potentially reducing the disparity in irisin levels in those with and without osteoporosis (59). To further explore the relationship between irisin and age, we conducted a meta-analysis of correlation coefficients between irisin and age in the included studies. The results revealed a summary r value of -0.18 (95% CI: -0.27 to -0.08), indicating a negative correlation between irisin and age (**Supplementary Figures 2**-**3**). Therefore, age may be a confounding factor for irisin levels. Further studies with diverse age groups and comparable ages between cases and controls are needed in the future.

Our study also highlighted the potential influence of menopause and sex hormone levels on the relationship between irisin and bone health. Several studies reported that the case groups had undergone menopause for a longer duration than the controls (13, 41). This discrepancy could result in lower estrogen levels and higher FSH levels, potentially contributing to osteoporosis. Previous research indicated that estradiol levels in healthy women were positively correlated with circulating irisin levels, suggesting that estradiol might modulate the relationship between irisin and OP (60). Although Colaianni et al. found that irisin serum levels positively correlate with bone mineral status in healthy children (61), our results on young women indicated no significant correlation between irisin and BMD. This finding could be attributed to the younger age when peak BMD had not yet been achieved. Puberty, a critical period for substantial bone growth, is highly influenced by external factors such as diet, physical activity, lifestyle, and medication (62). Additionally, hormones like estrogen may have stimulated bone formation in puberty (63). However, only six included studies reported the average age at menopause or the duration since menopause, and none reported sex hormone levels. This lack of data prevented us from analyzing these factors. Consequently, the utility of irisin as a biomarker appears to be more applicable in the postmenopausal population, where the impact of menopause on bone health is clearer. Further detailed studies on hormone levels are necessary to understand the relationship between irisin levels and bone health in premenopausal women, as well as in puberty and children populations, considering the complexities of hormonal influences and the transformation of bone health during these phases.

In the subgroup analysis based on BMI, no significant difference in irisin levels was observed between PMOP patients and non-PMOP controls among obese participants. A potential explanation was that irisin levels were negatively correlated with BMI, resulting in decreased irisin levels in obese populations (64, 65). However, current research on irisin concentrations and adiposity parameters remains controversial. Some studies have reported higher irisin levels in obese individuals compared to non-obese individuals (66, 67). Several factors may contribute to the observed discrepancies, such as decreased irisin sensitivity in white adipose tissue (WAT) or the potential dysfunction of irisin receptors. Consequently, our study suggested that irisin might serve as a predictor of osteoporosis primarily in non-obese populations. Further research with larger sample sizes and controls for relevant confounding factors is required to investigate the role of obesity in the relationship between irisin and osteoporosis.

Furthermore, our analysis revealed subtle variations in the decrease of irisin levels among postmenopausal women from different ethnic backgrounds. This variation could potentially be attributed to FNDC5 gene polymorphism among the diverse ethnic groups. Cetina et al. discovered an association between the rs3480 polymorphism of the FNDC5 gene and the risk of osteopenia at the total femoral and femoral neck in postmenopausal Mayan-Mestizo women (68). However, the scarcity of comprehensive genetic studies across various ethnicities underscores the need for further research in this area. Additionally, our study also found that the included Caucasian participants had higher BMIs than Asians, implying a potential interplay between BMI and ethnicity in influencing irisin levels.

Our meta-analysis also highlighted the impact of diverse definitions of OP and control groups used in the studies. While most of the included studies had a uniform standard for osteoporosis, defined as a T score below -2.5, the definition of the control group varied. Specifically, when control groups were defined as having a T-score below -1, this broader criterion led to a larger number of participants with lower BMD in the control group. This criterion reduced the overall T-score difference between the control and OP groups, potentially diminishing the diagnostic effectiveness of irisin as a biomarker. This observation emphasized the importance of establishing more consistent criteria in future research to elucidate the relationship between irisin levels and BMD more accurately.

Variations in publication language also presented differing outcomes regarding irisin levels, with the Chinese-language article showing decreased irisin levels in PMOP populations, whereas the English-language articles showed no significant differences. This disparity could be due to the inclusion of Asian populations in Chinese-language articles. In contrast, English-language studies encompassed a more diverse sample, including both Asian and Caucasian subjects. Despite these differences, the results of publication bias and sensitivity analysis demonstrated that there was no publication bias and the robustness of the pooled results. However, the results of the correlation between irisin and femoral BMD should be interpreted with caution because of potential publication bias. Consequently, future studies with larger sample sizes and inclusive of different populations are needed to enhance the applicability of irisin as a biomarker.

Another potential source of heterogeneity in our meta-analysis was the variety of irisin detection methods used. At least seven types of commercial ELISA kits were used, each with varying detection range and accuracy. At present, there is no uniform standard for detecting irisin, and some studies have raised concerns about the lack of required specificity of antibodies in existing commercial ELISA kits. Albrecht has advocated for the use of quantitative mass spectrometry with labeled peptides for more precise irisin quantification (69). Although this method is advanced, it requires multi-step sample preparation that may introduce uncontrollable variations in measurements. Despite the subgroup analysis did not identify specific ELISA kits as a source of heterogeneity, the discrepancies in the kits utilized in different studies represent a significant methodological heterogeneity that cannot be overlooked. Future research should focus on employing more reliable methodologies to accurately assess irisin levels and to reduce methodological heterogeneity between studies, thereby enhancing the reliability of the studies.

The inclusion of Chinese literature in this meta-analysis may raise concerns about the limited accessibility of these publications. In recent years, there has been a notable increase in research on irisin and bone health published in Chinese. However, these papers are not easily accessible to non-Chinese readers due to language barriers. Despite these challenges, it is a common practice to include Chinese articles in systematic reviews and meta-analysis. The inclusion of Chinese literature facilitates the understanding of a clinical issue at the global level. To improve the accessibility of Chinese literature, we have provided links to the Chinese databases and a detailed search strategy (**Supplementary File 1**). This approach could enhance the reproducibility of our systematic review. Furthermore, we have thoroughly reviewed and extracted the features and key data from the included literature in our study.

This systematic review and meta-analysis have several advantages. Firstly, we conducted a comprehensive search of both English and Chinese literature, ensuring a broad capture of available data and minimizing the risk of language bias. Furthermore, the enhanced cultural and geographic diversity of the data contributes to the global applicability of our findings. Secondly, our study included both postmenopausal and premenopausal populations, allowing for a more detailed analysis of menopausal status and its impact on the relationship between irisin and BMD. This approach ensures that our results are applicable to a broader demographic, providing insights into how irisin may be utilized in preventive strategies and treatments for osteoporosis at different stages of a woman’s life. Thirdly, we performed an extensive set of subgroup and meta-regression analysis to elucidate the heterogeneity of included studies. Finally, despite the significant heterogeneity in our findings, the quality of the included studies ranged from moderate to high, enhancing the overall reliability of our analyses. We also evaluated the quality of evidence for each outcome using the well-recognized GRADE scale.

Despite the novelty of our findings, this study has several limitations. First, the high heterogeneity within each subgroup suggested the presence of unknown factors that could contribute to the heterogeneity, such as the metabolic status, body composition, hormone levels, and lifestyle factors of the different populations studied. This significant heterogeneity led to serious inconsistency in the evidence quality assessment according to the GRADE guidelines, resulting in a very low-quality rating for each evidence. Therefore, the results of the present study should be interpreted with caution. Additionally, the impact of reduced physical activity and muscle mass following osteoporotic fractures on irisin levels merited consideration. The lack of prospective studies limited our ability to establish causality in the irisin-OP relationship. Moreover, the limited literature focusing on premenopausal women and the scarcity of detailed data on sex hormone levels in the included studies posed challenges in fully understanding the impact of sex hormones on the relationship between irisin levels and BMD.

## 5 Conclusion

In summary, our meta-analysis suggested that lower circulating irisin levels were associated with OP and fractures in postmenopausal women, with a positive correlation to BMD. These findings suggested the potential utility of circulating irisin levels as a biomarker for osteoporosis. However, due to the very low quality of the evidence, these results should be interpreted with caution. The incorporation of irisin assessment into routine clinical practice or combining irisin measurements with other diagnostic tools could improve early diagnosis and intervention strategies. This integration could lead to the development of individualized treatment plans to prevent or mitigate bone loss in populations at risk.

Further longitudinal studies and randomized controlled trials (RCTs) are necessary to validate the relationship between irisin levels and osteoporosis. Longitudinal studies would provide insight into the temporal relationship and causality between changes in irisin levels and bone density over time. RCTs could evaluate the efficacy of interventions aimed at modifying irisin levels, such as physical exercise or pharmacological treatments, in the prevention or treatment of osteoporosis. Additionally, studies investigating the molecular mechanisms by which irisin influences bone metabolism would further clarify its role and potential as a therapeutic target.

## Data availability statement

The original contributions presented in the study are included in the article/**Supplementary Material**. Further inquiries can be directed to the corresponding author.

## Author contributions

XS: Data curation, Formal analysis, Investigation, Methodology, Writing – original draft, Writing – review & editing, Visualization. YC: Investigation, Supervision, Writing – original draft, Writing – review & editing, Data curation. JZ: Methodology, Supervision, Writing – original draft, Writing – review & editing. MY: Data curation, Methodology, Writing – original draft, Writing – review & editing, Investigation. LH: Methodology, Writing – original draft, Writing – review & editing, Data curation. JL: Data curation, Writing – original draft, Writing – review & editing. LX: Conceptualization, Funding acquisition, Methodology, Supervision, Writing – original draft, Writing – review & editing.
