# Supplementary material for: The association between circulating irisin levels and osteoporosis in women: a systematic review and meta-analysis of observational studies
Source: Front Endocrinol (Lausanne). 2024 Aug 8;15:1388717. doi: 10.3389/fendo.2024.1388717 (PMC11338845; doi:10.3389/fendo.2024.1388717)
Supplement: Supplementary file 1 [file DataSheet_1.pdf]

Supplementary Material

The association between circulating irisin levels and osteoporosis in women: a systematic review and meta-analysis of observational studies

Xiaoyang Shen[1,2,3†], Yan Chen[1,2,3†], Jing Zhang [1,2,3], Meina Yang[1,2,3], Lu Huang [1,2,3], Jiaqi Luo[1,2,3], Liangzhi Xu[1,2,3]*

*** Correspondence:** Liangzhi Xu：xuliangzhi@scu.edu.cn

1 Supplementary File 1 Search strategy

PubMed (n=209)

#1 (irisin[Title/Abstract]) OR (FNDC5[Title/Abstract])

#2 (((((((((((((bone[Title/Abstract]) OR (osteoporosis[Title/Abstract])) OR (BMD[Title/Abstract])) OR (Bone Densities[Title/Abstract])) OR (Density, Bone[Title/Abstract])) OR (Bone Mineral Density[Title/Abstract])) OR (Bone Mineral Densities[Title/Abstract])) OR (Density, Bone Mineral[Title/Abstract])) OR (Bone Mineral Content[Title/Abstract])) OR (Bone Mineral Contents[Title/Abstract])) OR (Bone Density[MeSH Terms])) OR (BONE[MeSH Terms])) OR (osteoporosis[MeSH Terms])) OR (Fractures, Bone[MeSH Terms])) OR (Fracture[Title/Abstract])

#3 #1 AND #2

Embase (n=289)

#1 'bone density'/exp

#2 'osteoporosis'/exp

#3 'fracture'/exp

#4 'bone density':ab,ti

#5 'osteoporosis':ab,ti

#6 'fracture':ab,ti

#7 'bmd':ab,ti

#8 'bone'/exp

#9 'bone':ab,ti

#10 #1 OR #2 OR #3 OR #4 OR #5 OR #6 OR #7 OR #8 OR #9

#11 'fndc5':ab,ti

#12 'irisin':ab,ti

#13 #11 OR #12

#14 #10 AND #13

Web of Science (n=446)

1 "((((TS=(bone density)) OR TS=(osteoporosis)) OR TS=(fracture)) OR TS=(BMD)) OR TS=(bone)"

2 "(TS=(irisin)) OR TS=(FNDC5) "

3 "#1 AND #2"

Cochrane Library (n=5)

#1 MeSH descriptor: [Bone Density] explode all trees

#2 MeSH descriptor: [Osteoporosis] explode all trees

#3 (fracture):ti,ab,kw (Word variations have been searched)

#4 (bone density):ti,ab,kw (Word variations have been searched)

#5 (osteoporosis):ti,ab,kw (Word variations have been searched)

#6 MeSH descriptor: [Fractures, Bone] explode all trees

#7 #1 OR #2 OR #3 OR #4 OR #5 OR #6

#8 (irisin):ti,ab,kw

#9 (FNDC5):ti,ab,kw

#10 #8 OR #9

#11 #7 AND #10

China National Knowledge Infrastructure database（中国知网）(n=61)

Website：https://www.cnki.net/

(TKA=骨 OR TKA=骨密度 OR TKA=骨质疏松 OR TKA=骨折) AND (TKA=irisin OR TKA=FNDC5 OR TKA=鸢尾素)

VIP database （维普） (n=36)

Website：http://www.cqvip.com/

(M=骨 OR M=骨密度 OR M=骨质疏松 OR M=骨折) AND (M=irisin OR M=FNDC5 OR M=鸢尾素)

Wanfang database（万方）(n=172)

Website： https://w.wanfangdata.com.cn

(主题:(骨) or 主题:(骨质疏松 or 主题:(骨折 or 主题:(骨密度)))) and (主题:(irisin or 主题:(FNDC5 or 主题:(鸢尾素))))

2 Supplementary Figures and Tables

2.1 Supplementary Tables

Supplementary Table 1 Quality of the studies included in the systematic review and meta-analysis

Study	Year	**Selection**				**Comparability**	**Exposure**			Score
		Adequate case definition	Case representativeness	Selection of controls	Definition of controls	Comparability	Ascertainment of exposure	Same method of ascertainment	Non-response rate	
Maïmoun	2022	1	1	1	1	2	1	1	1	9
Zhu	2022	0	1	1	1	1	0	1	1	6
Zhou	2022	1	1	1	1	1	1	1	1	8
Liu	2021	1	1	1	1	1	1	1	1	8
Roomi	2021	1	1	0	1	1	1	1	1	7
Anastasilakis	2021	1	1	0	1	1	1	1	1	7
Zhang	2020	1	1	0	1	2	1	1	1	8
Shi	2019	0	1	0	1	1	1	1	1	6
Duan	2019	0	1	0	0	1	1	1	1	5
Park	2019	1	1	0	1	1	1	1	1	7
Yan	2018	1	1	0	1	1	1	1	1	7
Liang	2017	1	1	0	1	1	1	1	1	7
Engin-Üstün	2016	1	1	0	1	2	1	1	1	8
Palermo	2015	1	1	0	1	1	1	1	1	7
Anastasilakis	2014	1	1	0	1	1	1	1	1	7

Supplementary Table 2. Sensitivity analysis of outcomes.

Supplementary Table 2 (A). Sensitivity analysis of irisin levels in postmenopausal women with osteoporosis

Study omitted	Meta analysis results				Heterogeneity	
	SMD	95% CI		*P* value	I^2	*P* value
Roomi 2021	-1.40	-2.11	-0.69	<0.0001	98%	<0.00001
Liu 2021	-1.66	-2.51	-0.81	<0.0001	98%	<0.00001
Liang 2017	-1.77	-2.63	-0.92	<0.0001	98%	<0.00001
Engin-Üstün 2016	-1.79	-2.66	-0.92	<0.0001	98%	<0.00001
Palermo 2015	-1.78	-2.61	-0.95	<0.0001	98%	<0.00001
Anastasilakis 2014	-1.94	-2.68	-1.21	<0.00001	98%	<0.00001
Zhu 2022	-1.77	-2.76	-0.78	0.0004	98%	<0.00001
Zhou 2022	-1.47	-2.23	-0.71	0.0001	98%	<0.00001
Zhang 2020	-1.16	-1.86	-0.46	0.001	98%	<0.00001
Shi 2019	-1.76	-2.63	-0.90	<0.0001	98%	<0.00001
Duan 2019	-1.78	-2.61	-0.95	<0.0001	98%	<0.00001
Combined	-1.66	-2.43	-0.89	<0.0001	98%	<0.00001

Supplementary Table 2 (B). Sensitivity analysis of irisin levels in postmenopausal women with osteoporotic fractures

Study omitted	Meta analysis results				Heterogeneity	
	SMD	95% CI		*P* value	I^2	*P* value
Liu 2021	-1.41	-2.92	0.10	0.07	98%	<0.00001
Anastasilakis 2021	-1.67	-2.66	-0.69	0.0008	97%	<0.00001
Yan 2018	-1.41	-2.88	0.06	0.06	98%	<0.00001
Palermo 2015	-1.44	-2.54	-0.34	0.01	98%	<0.00001
Anastasilakis 2014	-0.44	-0.88	0.00	0.05	88%	<0.0001
Combined	-1.25	-2.15	-0.34	0.007	97%	<0.00001

Supplementary Table 2 (C). Sensitivity analysis of the relationship between irisin levels and the BMD

Study omitted	Meta analysis results				Heterogeneity	
	Fisher's Z	95% CI		*P* value	I^2	*P* value
Lumbar BMD						
Roomi 2021	0.31	0.16	0.46	<0.0001	88%	<0.00001
Park 2019	0.43	0.20	0.66	0.0002	95%	<0.00001
Yan 2018	0.43	0.20	0.66	0.0003	95%	<0.00001
Palermo 2015	0.44	0.22	0.66	0.0001	95%	<0.00001
Zhu 2022	0.38	0.12	0.64	0.004	95%	<0.00001
Zhou 2022	0.38	0.15	0.61	0.001	96%	<0.00001
Zhang 2020	0.39	0.15	0.63	0.002	96%	<0.00001
Liang 2017	0.43	0.20	0.66	0.0003	95%	<0.00001
Maïmoun 2022	0.43	0.21	0.65	0.0002	96%	<0.00001
Combined	0.40	0.19	0.61	0.0002	95%	<0.00001
Femoral BMD						
Park 2019	0.36	0.25	0.48	<0.00001	76%	0.004
Palermo 2015	0.35	0.22	0.48	<0.00001	83%	<0.00001
Zhu 2022	0.28	0.14	0.41	<0.0001	75%	0.0005
Liu 2021	0.29	0.12	0.45	0.0006	86%	<0.00001
Shi-ERALY[a] 2019	0.31	0.16	0.45	<0.0001	86%	<0.00001
Shi-LATE[b] 2019	0.30	0.16	0.45	<0.0001	86%	<0.00001
Liang 2017	0.30	0.15	0.46	0.0001	86%	<0.00001
Maïmoun 2022	0.35	0.22	0.48	<0.0001	83%	<0.00001

Combined	0.32	0.19	0.45	<0.00001	84%	<0.00001
Femoral Neck BMD						
Yan 2018	0.35	0.13	0.58	0.002	86%	<0.00001
Palermo 2015	0.37	0.16	0.57	0.0004	88%	<0.00001
Zhou 2022	0.28	0.08	0.49	0.007	88%	<0.00001
Zhang 2020	0.27	0.12	0.42	0.0006	73%	0.006
Liang 2017	0.33	0.08	0.57	0.009	89%	<0.00001
Maïmoun 2022	0.39	0.20	0.58	<0.0001	87%	<0.00001
Combined	0.33	0.15	0.52	0.0005	87%	<0.00001

Annotation: a: Early postmenopausal group (within 10 years after menopause); b: Late postmenopausal group (more than 10 years after menopause)

Supplementary Table 3 GRADE assessment for all outcomes evaluated in this systematic review meta-analysis

Certainty assessment							Relative Effect (95% CI)	Certainty	Importance
**No of studies**	**Study design**	**Risk of bias**	**Inconsistency**	**Indirectness**	**Imprecision**	**Other considerations**			
**irisin concentration in patients with PMOP**									
11	observational studies	not serious	serious[a]	not serious	not serious	none	SMD -1.66 (-2.43 to -0.89)	⨁◯◯◯ Very low	IMPORTANT
**irisin concentration in patients with fracture**									
5	observational studies	not serious	serious[a]	not serious	not serious	none	SMD-1.25 (-2.15 to -0.34)	⨁◯◯◯ Very low	IMPORTANT
**association of irisin concentration with lumbar BMD**									
9	observational studies	not serious	serious[a]	not serious	not serious	none	Fisher's Z 0.40 (0.19 to 0.61)	⨁◯◯◯ Very low	IMPORTANT
**association of irisin concentration with femoral BMD**									
7	observational studies	not serious	serious[a]	not serious	not serious	suspected reporting bias[b]	Fisher's Z -0.32 (0.19 to 0.45)	⨁◯◯◯ Very low	IMPORTANT
**association of irisin concentration with femoral neck BMD**									
6	observational studies	not serious	serious[a]	not serious	not serious	none	Fisher's Z 0.33 (0.15 to 0.52)	⨁◯◯◯ Very low	IMPORTANT

Abbreviations: CI, confidence interval; SMD, standardized mean difference

a Serious inconsistency due to significant heterogeneity ($I^2 > 50\%$). b Egger's test $P < 0.05$.

2.2 Supplementary Figures

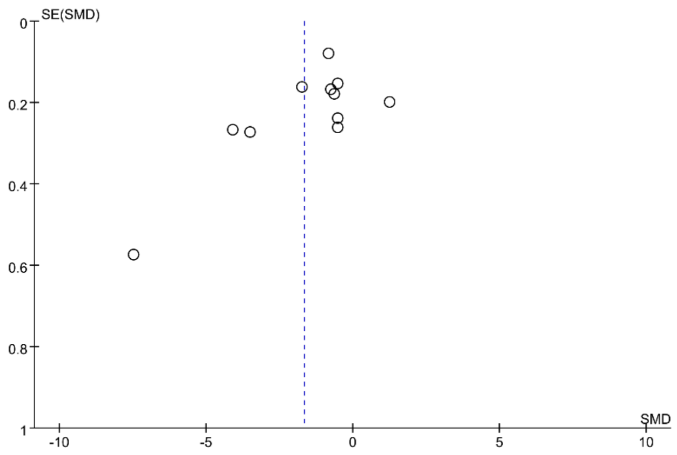

(A)

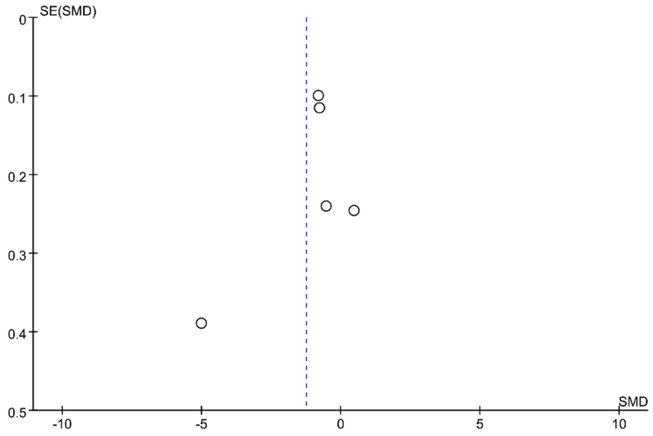

(B)

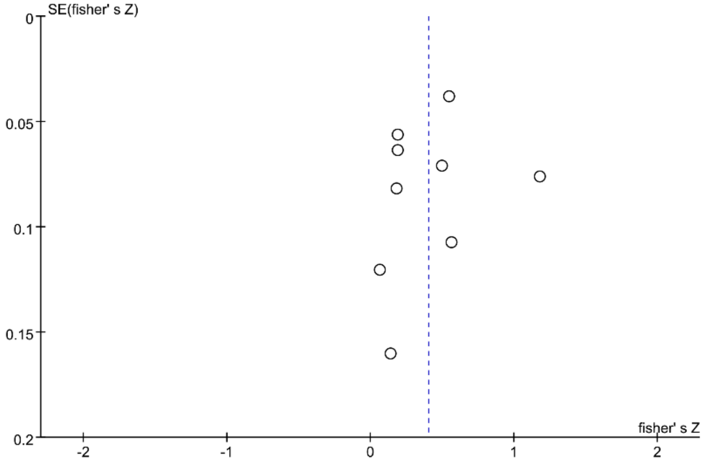
SE(fisher' s Z)
0
0.05
0.1
0.15
0.2
fisher' s Z
-2
-1
0
1
2

(C)

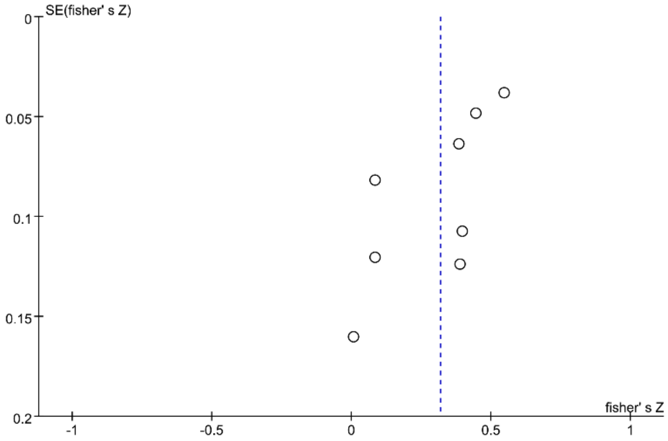
SE(fisher' s Z)
0
0.05
0.1
0.15
0.2
fisher' s Z
-1
-0.5
0
0.5
1

(D)

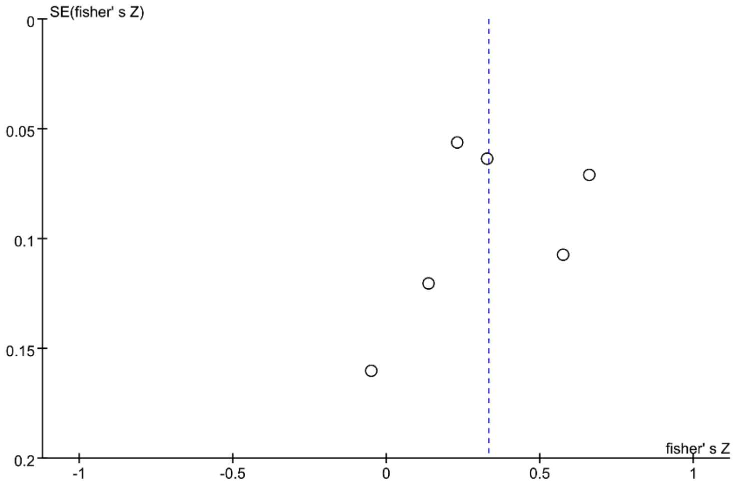

(E)

Supplementary Figure 1. Funnel plot of included studies.

(A)Funnel plot of the irisin levels in the PMOP group versus the non-PMOP group. (B) Funnel plot of the irisin levels in postmenopausal women with osteoporotic fractures versus the non-fracture group. (C) Funnel plot of the correlation coefficient between the irisin levels and lumbar BMD. (D) Funnel plot of the correlation coefficient between the irisin levels and femoral BMD. (E) Funnel plot of the correlation coefficient between the irisin levels and femoral neck BMD.

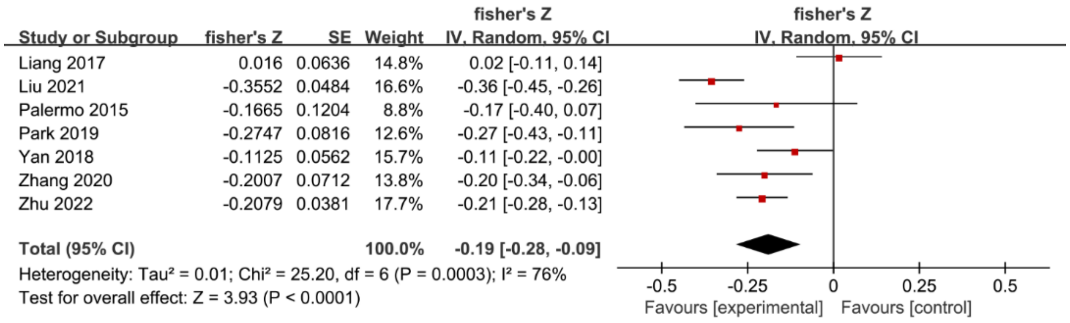

Supplementary Figure 2. Forest plot of meta-analysis of the correlation coefficient between the irisin levels and age

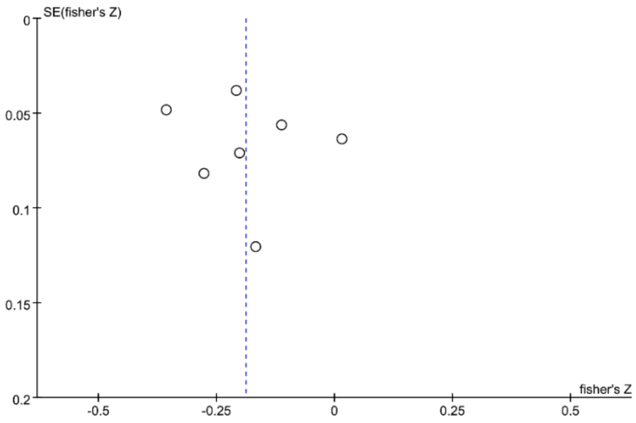

Supplementary Figure 3. Funnel plot of the correlation coefficient between the irisin levels and age.
